# The Role of Comorbid Symptoms in Perceived Stress and Sleep Problems in Adolescent ADHD

**DOI:** 10.1007/s10578-022-01320-z

**Published:** 2022-01-30

**Authors:** Matilda A. Frick, Jenny Meyer, Johan Isaksson

**Affiliations:** 1 Department of Medical Sciences, Child and Adolescent Psychiatry Unit, Uppsala University, Uppsala, Sweden; 2 Department of Psychology, Division of Emotion Psychology, Uppsala University, Uppsala, Sweden; 3 Center of Neurodevelopmental Disorders (KIND), Centre for Psychiatry Research, Department of Women’s and Children’s Health, Karolinska Institutet & Stockholm Health Care Services, Region Stockholm, Stockholm, Sweden

**Keywords:** Comorbid symptoms, Attention-deficit/hyperactivity disorder (ADHD), Perceived stress, Sleep problems

## Abstract

We examined perceived stress and sleep problems in adolescent ADHD and whether this varies as a function of ADHD presentation and sex. Further, we mapped structural associations between ADHD symptoms, comorbid symptoms, perceived stress, and sleep problems. Participants were 306 adolescents aged 13–19 years (66.8% females, 193 had an ADHD diagnosis, 113 were controls). Parents rated ADHD symptoms, all other constructs were self-rated. Adolescents with ADHD had elevated levels of perceived stress and sleep problems. Girls with ADHD reported the highest levels of perceived stress. Emotional symptoms mediated the effect of inattention whereas conduct problems mediated the effect of hyperactivity/impulsivity on stress and sleep. Perceived stress and sleep problems should be considered when mapping ADHD-related problems. Comorbid symptoms are potential intervention targets that may increase treatment response.

## Introduction

Adolescence is a period marked by increases in health-related problems such as perceived stress and sleep disturbances [1–3]. Individuals with attention-deficit/hyperactivity disorder (ADHD) may by particularly vulnerable for developing such health problems [4, 5]. ADHD is one of the most prevalent psychiatric conditions, affecting 5–7% of children and adolescents worldwide [6] and is characterized by elevated and disabling symptoms of inattention and hyperactivity/impulsivity [7]. There are three different presentations of the condition, each marked by a specific pattern of symptoms [7]. The combined presentation (ADHD-C) with symptoms of both domains, the predominantly inattentive presentation (ADHD-I), and the predominantly hyperactive/impulsive presentation (ADHD-H). Comorbidity rates are high, with up to 50% of children with ADHD having a comorbid externalizing disorder, such as oppositional defiant disorder or conduct disorder [8] and up to 40% presenting emotional symptoms such as depression or anxiety [9]. Further, 50–70% experience difficulties in peer relationships [10] and an estimated 25–70% sleep problems [11]. Previous findings indicate that perceived stress is elevated in adolescents with ADHD [4], but the evidence is still scarce. For many individuals with the condition, the associated problems with perceived stress, sleep problems, and comorbid symptoms add substantially to the difficulties in daily functioning.

Stress concerns an alarm response to novel or threatening situations resulting in arousal within the organism [12]. The stimulus that evokes the stress response is referred to as a stressor. There are large individual differences in how individuals perceive and respond to a stressor, which is influenced by previous experiences and expectations of the outcome [12]. Levels of perceived stress and emotional symptoms usually increase during adolescence, and are especially pronounced in female adolescents [13]. Previous studies on stress in ADHD have mainly been conducted in adult samples (e.g., [14–16]). Although the literature is limited, it is proposed that adolescents with ADHD in general and females with ADHD in particular are at risk for elevated levels of perceived stress [4]. Further, ADHD is associated with a higher exposure of stressors, and perceived stress has been found to be associated with comorbid emotional and externalizing symptoms in individuals with ADHD [17]. A recent qualitative study reported that adolescents with ADHD experienced stress as closely intertwined with negative feelings and anxiety [18]. As such, emotional symptoms may play a specific role in the elevated levels of stress in ADHD. In adults, inattentive symptoms have been a more consistent predictor of stress, as opposed to hyperactive/impulsive symptoms [14]. Positive peer relations can be a source of protection against negative effects of stressors [19, 20], while peer problems can be considered a stressor in itself [21]. To our knowledge, the contribution of symptom domains and ADHD presentation to levels of perceived stress has not been targeted in adolescents and the mediating role of comorbid symptoms needs further investigation.

There is an established connection between high levels of perceived stress and poor sleep [22, 23]. Sleep problems include for instance bedtime resistance, delayed sleep onset, fear of sleeping alone, sleep walking, shortened sleep time, frequent awakenings, and non-restorative sleep [11]. Sleep problems may have a profound impact on memory, learning as well as on emotional and cognitive processing [11]. Adolescence is a period characterized by alternations and maturation of the brain, reduced parental monitoring, and competing social demands, which together result in dramatic shifts in sleep behaviors that may lead to less and insufficient sleep [24–26]. Sleep problems is an important feature of ADHD, with up to threefold higher incidence compared to typically developed children (25–70% vs 7–20%) [5, 11]. ADHD presentation has been suggested as important for the occurrence of sleep problems. In a previous study, ADHD-C was associated with the highest levels of problems whereas the inattentive presentation did not differ from controls [27]. However, it is not clear whether the associated sleep problems are a core feature of ADHD resulting from neurobiological underpinnings, or resulting from comorbidity [11]. For instance, one study found that stimulant medication as well as comorbid internalizing and disruptive disorders accounted for the association between ADHD and sleep problems [28]. As such, it is important to understand the mechanisms underlying sleep problems in ADHD. Further, the majority of studies on sleep problems in ADHD have been conducted in predominately male samples (e.g., [28, 29]) and any sex-specific effect on the association remains to be decided. Findings also suggest that comorbid emotional symptoms increased sleep problems whereas externalizing symptoms did not [27, 30]. Yet, both studies involved mostly younger children (mean age ~ 9 years), and in the study by Becker and colleagues [30] all constructs were parent rated, which may imply rater bias. These findings are inconclusive as others have found associations between conduct problems and poor sleep [31]. In addition, peer problems have positive associations with sleep problems [32, 33], and may as such contribute to increased levels beyond other comorbid constructs.

Manageable stress levels and adequate sleep are important for wellbeing and may worsen daily functioning if poor. The associations between ADHD and perceived stress and sleep problems are complex and warrant further examination [34–36]. From the literature, it is not clear whether elevated perceived stress and sleep problems arise from the core ADHD symptoms or from comorbid problems (i.e., peer problems, emotional symptoms, and conduct problem). Mapping these associations will guide the search for tailored interventions. To our knowledge, these relations have not been examined in adolescents with ADHD, taking the effects of ADHD presentation and sex into consideration. Thus, to rectify these limitations, we aim to investigate perceived stress and sleep problems in adolescent ADHD with a specific focus on the role of ADHD presentation and comorbid symptoms, taking potential sex differences into account. Within our line of research examining feasibility and efficacy of psychological treatments for adolescents with ADHD comprising samples enriched with females with and without ADHD [37, 38], we saw an opportunity to approach these research questions.

### Aims and Hypotheses

1. We examined if adolescents with ADHD experienced more perceived stress and sleep difficulties compared to peers without ADHD, and whether this varied as a function of ADHD presentation and sex. We expected to find higher levels of perceived stress and sleep difficulties in the ADHD group, and that levels would be more elevated in girls compared to boys in both groups.
2. We mapped the structural associations between ADHD symptoms, comorbid symptoms (peer problems, emotional symptoms, and conduct problems), perceived stress, and sleep difficulties to examine whether comorbid symptoms mediated the associations between ADHD symptoms and perceived stress and sleep. Sex was examined as covariate and as potential moderator.

## Method

### Procedure and Participants

Participants were 306 adolescents aged 13–19 years (females, n = 203, 66.8%), of which 193 had an ADHD diagnosis (females, n = 124, 64.9%) and 113 were recruited as controls (females, n = 74, 70.5%). In the control group, eight reported either a diagnosis of ADHD (n = 2) and/or symptom levels above the cut-off for ADHD (see below; n = 7). Accordingly, we excluded these individuals from group comparisons but kept them in the dimensional analyses, hence the final control group constitute n = 105. Only one individual in the ADHD group was characterized as predominantly hyperactive/impulsive. Hence, no group comparisons regarding this presentation were conducted.

Participants with ADHD comprised two different sub samples (S1 and S2), both recruited via Child and Adolescent Psychiatric (CAP) units in Sweden (i.e. both rural and urban). S1 constituted 164 adolescents aged 15–18 years, recruited to a randomized controlled trial (RCT) examining effects of psychotherapy [38] while S2 constituted 29 adolescents aged 13–18 years recruited to larger cohort, of which the younger participants partook in a feasibility study [37]. The control sample (S3; aged 14–19 years) was recruited via local schools as a reference group. Of the ADHD group, 68.9% (n = 133) received prescribed ADHD medication (e.g. Concerta, Elvanse, Strattera, or Intuniv). Exclusion criteria for recruitment to S1 were severe depression, suicidality, psychosis, bipolar disorder without stable medication, intellectual disability, brain injury, autism, or on-going substance abuse. Of the final sample in S1 59.9% had symptom levels indicating psychiatric comorbidity based on the strength and difficulties questionnaire (SDQ; [38]). For S2, no exclusion criteria were applied at recruitment and parents reported that 14 children (48.3%) had comorbid diagnoses (post-traumatic stress disorder n = 1, dyslexia n = 3, social phobia n = 2, depression n = 2, autism n = 1, medicated bipolar disorder n = 2, oppositional defiant disorder n = 3). Removing the child with a reported diagnosis of autism did not change the results and therefore the full S2 sample was used in the final analyses. None in the final S3 sample reported any psychiatric comorbidity. Ethical approval was obtained from the regional ethics board in Uppsala, Sweden (Dnr. 2020-05009). Parents and adolescents gave written informed consent to participate. For participation the adolescents received one (S2 and S3) or two (S1) movie tickets (worth approximately 10 USD each).

### Measures

#### Diagnostic Status

Diagnostic status, including ADHD presentation, was obtained from medical records (S1) or reported by parents (S2 and S3). Additionally, in S1 all participants were interviewed with a structured diagnostic interview performed by clinical psychologists (MINI-kid; [39]). For the purpose of the current study, due to these differing diagnostic methods and that some assessments were conducted in previous years, ADHD presentation was based on dichotomized cut-off points using the Adult ADHD Adolescent version, Parent-Report Scale, (ASRS-AP) as recommended by Kessler [40] and validated in a Swedish population [41]. That is, a symptom was considered as endorsed if shadowed in the ASRS-AP questionnaire, which corresponds to a symptom rating of 3 (i.e., “often”), 4 (i.e., “always”), or in a few instances a rating of 2 (i.e., “sometimes”). Further, endorsement of six or more symptoms (five or more for age 17 and older, in accordance with the DSM-5) from each symptom domain respectively (i.e., inattention and hyperactivity/impulsivity) informed on ADHD presentation (i.e., ADHD-C, ADHD-I, and ADHD-H). Participants who had previously been diagnosed with ADHD, but currently endorsed fewer symptoms of both symptom domains were classified as ADHD-not other specified (NOS). Diagnostic sensitivity analyses using *χ*^2^ cross tabulation showed that diagnostic status according to guidelines by Kessler and colleagues [40] was significantly correlated with clinical diagnoses (S1; Pearson’s *χ*^2^ = 13.62, *p* = 0.034) and parent-reported diagnostic status (S2 and S3; Pearson’s *χ*^2^ = 137.96, *p* < 0.0001).

#### Symptom Measures

To reduce rater bias, parents reported on ADHD symptoms and adolescents reported on comorbid symptoms, perceived stress, and sleep problems. This was informed by studies suggesting that parents are well-suited to rate ADHD symptoms in their adolescents [42] and that adolescent reports are more sensitive to subjective experiences of mental health problems [43]. The ratings were conducted before the adolescents participated in the psychological treatments for which they were recruited.

##### ADHD Symptoms

Parents reported on adolescents’ symptoms of inattention and hyperactivity/impulsivity using the ASRS-AP [40]. The scale consists of 18 items on a scale from 0 to 4, of which nine correspond to the diagnostic criteria for inattention and nine to hyperactivity/impulsivity. We used the mean of each subscale as an index of inattention and hyperactivity/impulsivity respectively. The scale has shown good diagnostic validity in a Swedish sample [41, 44]. Cronbach’s alpha in the current sample was α = 0.95 for inattention and α = 0.93 for hyperactivity/impulsivity.

##### Comorbid Symptoms

The adolescents reported on comorbid symptoms using the subscales peer problems, emotional symptoms, and conduct problems from the SDQ [45]. Each subscale comprises five items on a scale ranging from 0 to 2. We used the mean of each subscale to index the respective comorbid domain. The scale has shown adequate validity in Swedish adolescents [46]. Cronbach’s alpha in the current sample was α = 0.59 for peer problems, α = 0.73 for emotional symptoms, and α = 0.62 for conduct problems.

##### Stress

The adolescents reported levels of perceived stress using the Pressure Activation Stress (PAS) scale [4, 47]. The PAS scale constitutes of 11 items on a scale from 0 to 4, of which seven items corresponds to a dimension capturing pressure and four items to a dimension capturing activation. The PAS has shown good external validity in a Swedish sample of children with and without ADHD [4]. We used the mean across both dimensions as a global index of perceived stress. Cronbach’s alpha in the current sample was α = 0.86.

##### Sleep

The adolescents reported on sleep difficulties using the Karolinska sleep questionnaire (KSQ; [48]).The KSQ constitutes of seven items on a scale from 0 to 5 about sleep difficulties. Four items concern sleep quality and three items concern awakenings. We used the mean across both dimensions as a global index of sleep difficulties. The KSQ has shown good validity [48]. Cronbach’s alpha in the current sample was α = 0.85.

### Analytic Strategy

IBM SPSS Statistics 26 was used for preliminary analyses and analysis of group differences (Aim 1). The lavaan package [49] in R was used for the path analysis (Aim 2). We converted data to *z* scores to screen for outliers (> 3) and examined skewness and kurtosis using guidelines provided by George and Mallery [50]. To handle missing data, the possibility of imputing missing values was scrutinized for Aim 1 and maximum likelihood [51] was used for Aim 2. We used *t* tests, one-way ANOVAs, and post-hoc tests (i.e., Tukey HSD and Games-Howell) to examine group differences based on diagnostic status and ADHD presentation. We conducted a two-way ANOVA to examine interaction effects between group and sex, controlling for age. To confirm the theoretical associations between the constructs of interest, we conducted bivariate correlations using Spearman’s *Rho* for ordinal variables and Kendall’s *Tau* for nominal data (i.e. sex). After that, structural associations between symptoms were examined using multi-mediation full path analysis [52]. Sex and age were explored as covariates. We present regression parameters and *R*^2^ to depict explained variance. In a separate full path model, sex was explored as moderator in that interaction terms between sex and all predictors were included and then excluded in a stepwise procedure in which we step-by-step excluded the least significant interaction term [53]. *P* values < 0.05 were regarded as significant.

All hypotheses and analyses were preregistered in the open science framework (OSF) prior to analyses (https://osf.io/rvu8x/?view_only=2ef66b54adb0451ab4e6e5ec49586b3e), with two minor deviances for Aim 2. First, the path model had 0 degrees of freedom and was as such fully saturated with perfect fit. Hence, model fit statistics are not used or reported. Instead, we report and base our interpretation of the model on the regression parameters. Second, our initial aim was to compare the current model to a model with stress and sleep as mediators. However, as both models were equally strong we chose to focus on the one with our main constructs (stress and sleep) as endogenous variables.

## Results

### Preliminary Results

See Table 1 for descriptive statistics and Table 2 for correlations between all study variables. We found no outliers. Skewness ranged from − 0.605 to 0.854 and kurtosis ranged from − 1.050 to 0.678, which were considered acceptable for use of parametric tests [50]. Missing data constituted 5.27%. Little’s MCAR test was non-significant (*χ*^2^ = 15.28, DF = 16, *p* = 0.505), which indicates that data was missing completely at random and that multiple imputation can be used. Five sets of imputations were made using standard fully conditional specification (FCS-Standard; [54]). See Table 2 for correlations between the study variables. In the ADHD group, individuals with medication had significantly lower levels of inattention compared to non-medicated individuals (M = 2.80 vs 3.16, *p* < 0.0001), but they did not differ regarding hyperactivity/impulsivity, perceived stress, or sleep problems (*p*s > 0.401). For S2 and S3, we have socio-demographic data on completed parental education (1 = elementary school, 2 = high school, 3 = university), living conditions (1 = cohabiting parents, 2 = separated parents), and parental birthplace (1 = both born in Scandinavia, 2 = one parent born in Scandinavia, 3 = both parents born outside of Scandinavia; see Table 1). The groups differed significantly on parental education (i.e., the mean across both parents, *p* = 0.008) but not on living conditions (Pearson’s *χ*^2^ = 0.092).

**Table 1:** Descriptive statistics

	Total sample		ADHD group				Control group			
			All		Males	Females	All		Males	Females
	n	Mean (SD)	n	Mean (SD)	Mean (SD)	Mean (SD)	n	Mean (SD)	Mean (SD)	Mean (SD)
Age	306	16.44 (1.13)	193	16.34 (1.17)	16.42 (1.22)	16.33 (1.12)	105	16.58 (1.03)	17.00 (0.95)	16.41 (1.02)
Sleep problems	288	2.13 (1.11)	180	2.34 (1.12)	2.24 (1.06)	2.41 (1.16)	103	1.68 (0.93)	1.67 (0.82)	1.68 (0.98)
Perceived stress	288	1.99 (0.79)	180	2.11 (0.82)	1.71 (0.75)	2.35 (0.77)	103	1.75 (0.68)	1.64 (0.55)	1.80 (0.72)
Inattention	287	2.22 (1.14)	191	2.89 (0.62)	2.83 (0.56)	2.91 (0.64)	88	0.77 (0.56)	0.86 (0.59)	0.73 (0.55)
Hyperactivity/impulsivity	287	1.55 (1.06)	191	2.05 (0.86)	2.01 (0.75)	2.06 (0.92)	88	0.45 (0.45)	0.49 (0.50)	0.44 (0.44)
Peer problems	288	0.48 (0.35)	180	0.53 (0.36)	0.50 (0.39)	0.55 (0.34)	103	0.37 (0.32)	0.35 (0.36)	0.38 (0.31)
Emotional symptoms	288	0.92 (0.52)	180	0.96 (0.51)	0.68 (0.47)	1.12 (0.46)	103	0.82 (0.51)	0.60 (0.51)	0.91 (0.49)
Conduct problems	288	0.49 (0.38)	180	0.60 (0.39)	0.55 (0.40)	0.63 (0.38)	103	0.28 (0.25)	0.35 (0.31)	0.25 (0.22)
ADHD-C			76	39.4%	35.8%	41.1%				
ADHD-I			87	45.1%	50.7%	41.2%				
ADHD-NOS			27	14.0%	11.9%	15.3%				
ADHD-H			1	0.5%	1.5%	0%				
Parental education			29	2.31 (0.63)			88	2.66 (0.44)		
Living conditions			29	Cohabit n = 17 (58.6%)			88	Cohabit n = 66 (75%)		
Parental birth county			29	Both Scandinavian n = 26 (89.7%) One Scandinavian n = 3 (10.3%)			88	Both Scandinavian n = 68 (77.3%) One Scandinavian n = 9 (10.2%) None Scandinavian n = 11 (12.5%)		

*C* combined presentation, *I* inattentive presentation, N*OS* not other specified, *H* hyperactive/impulsive presentation

**Table 2:** Correlations between study variables

	Age	Sex^a^	Sleep	Stress	Inattention	Hyp/Imp	PP	ES	CP
Age	1	− 0.095^*^	0.033	0.012	− 0.119^*^	− 0.156^**^	0.036	− 0.076	− 0.047
Sex (1 = boy, 2 = girl)^a^		1	0.002	0.220^***^	− 0.002	− 0.019	0.058	0.297^***^	0.000
Sleep			1	0.550^***^	0.368^***^	0.348^***^	0.272^***^	0.428^***^	0.384^***^
Stress				1	0.300^***^	0.319^***^	0.355^***^	0.515^***^	0.409^***^
Inattention					1	0.781^***^	0.184^**^	0.155^*^	0.530^***^
Hyp/Imp						1	0.218^***^	0.124^*^	0.585^***^
PP							1	0.388^***^	0.229^***^
ES								1	0.241^***^
CP									1

*Hyp/Imp* hyperactivity/impulsivity; *PP* peer problems; *ES* emotional symptoms; *CP* conduct problems

*p < 0.05; **p < 0.01, ***p < 0.001

^a^Kendall’s *Tau*, all other analyses conducted with Spearman’s *Rho*

### Group Differences

**Fig. 1:**
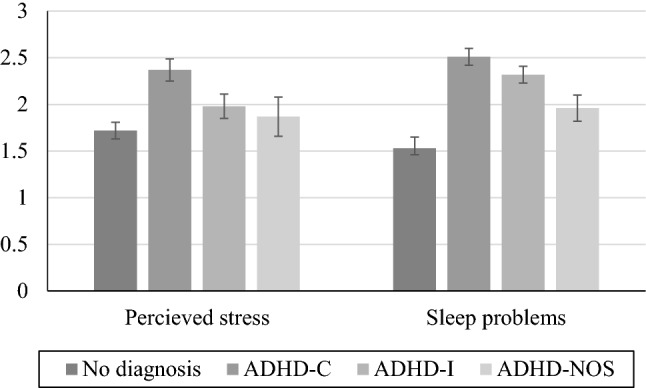
Perceived stress and sleep problems as a function of diagnosis and diagnostic presentation. *ADHD-C* combined presentation, *ADHD-I* inattentive presentation, *ADHD-NOS* not other specified. Standard errors in bars

**Fig. 2:**
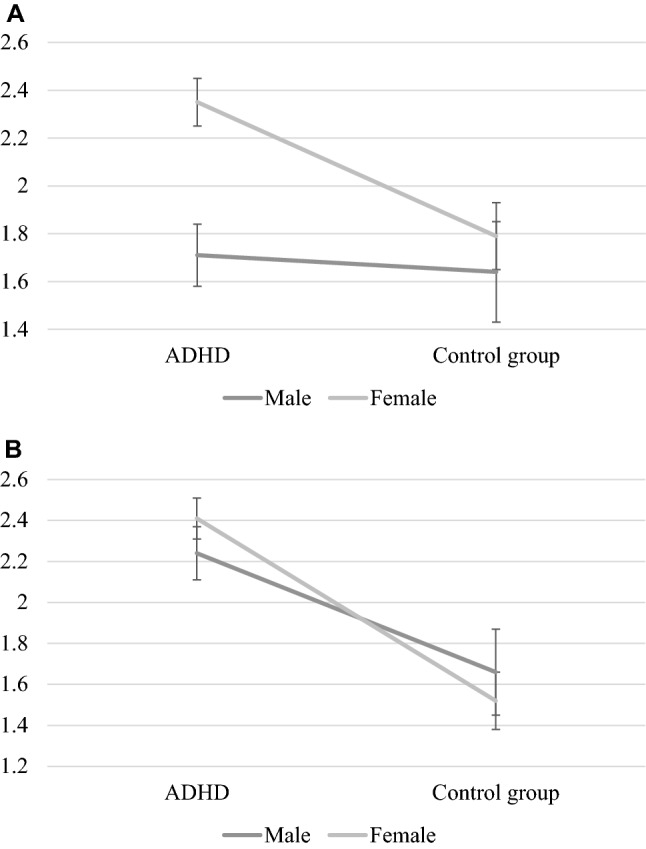
**a** Perceived stress as a function of diagnosis and sex. Standard errors in bars. **b** Sleep problems as a function of diagnosis and sex. Standard errors in bars

Adolescents with ADHD experienced more perceived stress (*t*(468) = 3.88, *p* < 0.0001) and sleep problems compared to their typically developed peers (*t*(1593) = 5.65, *p* < 0.0001). This varied as a function of ADHD presentation for both stress (*F*(3,257) = 9.37, *p* < 0.0001) and sleep (*F*(3,257) = 13.62, *p* < 0.0001). Specifically, ADHD-C reported significantly higher levels of perceived stress that the control group and the other ADHD presentations (*p*s = 0.02 to < 0.0001) and more sleep problems than the control group (*p*s < 0.0001; see Fig. 1). Further, also ADHD-I had significantly more sleep problems than the control group (*p*s < 0.0001; see Fig. 1). No other differences were significant. We found one significant interaction effect, in that girls with ADHD had the highest levels of perceived stress *F*(1,258) = 4.83, *p* = 0.029 (see Fig. 2a). No such interaction effect was found in relation to sleep problems, *F*(1,258) = 1.10, *p* = 0.296 (see Fig. 2b).

### Multi-Mediation Full Path Analysis

**Fig. 3:**
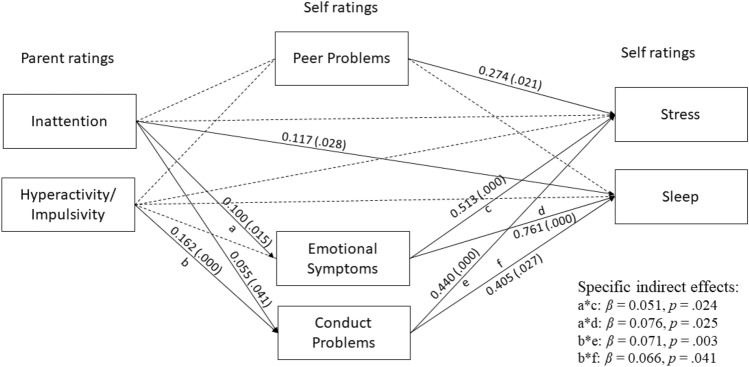
Path diagram for the multi-mediation full path model with depiction of statistically significant standardized estimates for direct and indirect effects. *P* values in parenthesis

Age was uncorrelated to all endogenous variables and hence not included in the model. See Fig. 3 for depiction of all significant paths and indirect effects and Tables 3 and 4 for presentation of all effects. Inattention had a direct effect on emotional symptoms, conduct problems, and sleep problems. Hyperactivity/impulsivity had a direct effect on conduct problems only. All comorbid constructs had direct effects on perceived stress. Emotional symptoms and conduct problems had direct effects on sleep problems. Emotional symptoms mediated the effect of inattention on perceived stress and sleep problems whereas conduct problems mediated the effect of hyperactivity/impulsivity on stress and sleep. The model explained 38.5% of the variation in perceived stress and 30.1% in sleep problems. Note in Table 3 that sex had significant effects on perceived stress and sleep problems, in that being female was associated with higher levels of perceived stress and being male was associated with more sleep problems when controlling for symptoms of ADHD and comorbidity. Sex did not moderate the relations between ADHD symptoms and the endogenous variables as no interaction term was significant, using the stepwise procedure.

**Table 3:** Depiction of all effects in the full path model

	Perceived stress			Sleep problems			Peer problems			Emotional symptoms			Conduct problems		
	*β*	*p*	*R*^*2*^	*β*	*p*	*R*^*2*^	*β*	*p*	*R*^*2*^	*β*	*p*	*R*^*2*^	*β*	*p*	*R*^*2*^
			0.385			0.301			0.054			0.147			0.335
Inattention	− 0.012	0.821		0.177	**0.028**		0.043	0.154		0.100	**0.015**		0.055	**0.041**	
Hyperactivity/Impulsivity	0.113	0.068		0.056	0.540		0.035	0.282		0.023	0.609		0.162	**0.000**	
Peer Problems	0.274	**0.021**		0.163	0.356										
Emotional Symptoms	0.513	**0.000**		0.761	**0.000**										
Conduct Problems	0.440	**0.000**		0.405	**0.027**										
Sex	0.261	**0.002**		− 0.255	**0.044**		0.045	0.272		0.371	**0.000**		0.034	0.401	

Significant values in bold

**Table 4:** Parameters for specific indirect effects

	*β*	Std. Error	*p*
Inattention → PP → Stress	0.012	0.010	0.225
Inattention → ES → Stress	**0.051**	0.023	**0.024**
Inattention → CP → Stress	0.024	0.014	0.076
Hyp/Imp → PP → Stress	0.010	0.010	0.330
Hyp/Imp → ES → Stress	− 0.012	0.023	0.610
Hyp/Imp → CP → Stress	**0.071**	0.024	**0.003**
Inattention → PP → Sleep	0.007	0.009	0.439
Inattention → ES → Sleep	**0.076**	0.034	**0.025**
Inattention → CP → Sleep	0.022	0.015	0.134
Hyp/Imp → PP → Sleep	0.006	0.008	0.484
Hyp/Imp → ES → Sleep	-0.017	0.034	0.610
Hyp/Imp → CP → Sleep	**0.066**	0.032	**0.041**
Sum of indirect effects	**0.316**	0.073	**0.000**
Total effects	**0.651**	0.090	**0.000**

Significant effects in bold

*Hyp/Imp* hyperactivity/impulsivity, *PP* peer problems, *ES* emotional symptoms, *CP* conduct problems

## Discussion

In the present study, we examined perceived stress and sleep problems among adolescents with ADHD with a special focus on ADHD presentation, sex, and the role of comorbid symptoms. We found elevated levels of perceived stress and sleeping difficulties in individuals with ADHD compared to their typically developed peers. Specifically, individuals with the combined presentation (ADHD-C) reported the highest levels of perceived stress and sleep difficulties, whereas the inattentive presentation (ADHD-I) had significantly more sleep problems that the control group. Girls with ADHD had the highest levels of perceived stress, whereas no such interaction effect for ADHD by sex was found for sleep problems. A multi-mediation full path model showed that emotional symptoms mediated the effect of inattention on perceived stress levels and sleep problems whereas conduct problems mediated the effect of hyperactivity/impulsivity on stress and sleep. The results suggest an intricate and specific interplay between ADHD symptom domain and comorbid symptoms in the presentation of self-rated perceived stress and sleep problems. Tolerable stress levels and restorative sleep are of great importance for daily functioning and wellbeing. It is therefore important to understand mechanisms underlying deviant perceived stress and sleep. As such, the results from the current study may guide the search for tailored treatment and interventions.

### Group Differences

Elevated stress and sleep problems are common during adolescence. Our results confirm our hypotheses and previous findings, that adolescents with ADHD are at increased risk for exposure to these health-related issues [1, 4]. Our findings regarding the effect of ADHD presentation are partly in contrast with previous results. Mayes and colleagues found that children with the combined presentation had the highest risk of sleep problems [27], whereas we found that both ADHD-C and ADHD-I were at increased risk. Further, Combs and colleagues found that inattention was the most consistent predictor of high levels of perceived stress [14], whereas we found elevated stress levels in ADHD-C only. Of note, none of these prior studies examined adolescents, which highlights the possibility that health-related risks may be different across the lifespan. The increased risk of ADHD-C could reflect a dose–response effect, in that individuals with the combined presentation in total have more symptoms than individuals with the other ADHD presentations. However, this is in part contradicted by the fact that individuals with the inattentive presentation had similar levels of sleep problems as ADHD-C. Rather, the results could reflect that each symptom domain have a specific contribution to increased stress levels and sleep problems, which is indicated by the pattern of mediated effects detected in the path analysis (see discussion below).

### Sex and Age

Interaction effects indicate that the higher perceived stress levels in the ADHD group are driven by elevated stress among adolescent girls with ADHD. This is in line with our hypothesis, corroborates previous findings [4], and underscores the need to monitor these symptoms carefully in this patient group. The pressure put on adolescents in general and on females in particular to perform well in school, at home, and socially may be an especially heavy burden for girls with ADHD. Interestingly, no such sex effect was found in relation to sleep problems. However, using a dimensional perspective sex had independent effects on both perceived stress and sleep problems, in that being female was associated with higher levels of perceived stress and being male was associated with more sleep problems. A potential mechanism could be that everyday strains manifest different for males and females. Where girls feel pressured and go at full speed to try to cope with academic and social demands, boys may for other reasons, such as staying up too late playing video games, not get enough sleep [55]. Interestingly, while the sex effect on stress seems driven by girls with ADHD, the effect of male sex on sleep problems seems driven by slightly increased levels of sleep problems in boys without ADHD, but this calls for further studies. Age was unrelated to comorbid symptoms, stress, and sleep, indicating that these problems are manifest early in adolescence and do not seem to escalate later in adolescence.

### Structural Associations and Mediating Effects

As for structural associations, inattentive symptoms had a direct effect on emotional symptoms, conduct problems, and sleep problems whereas hyperactivity/impulsivity had a direct effect on conduct problems only. All comorbid constructs had independent effects on perceived stress while emotional symptoms and conduct problems had independent effects on sleep problems. Of most interest for the sake of the current study, emotional symptoms mediated the effect of inattention on stress and sleep whereas conduct problems mediated the effect of hyperactivity/impulsivity on stress and sleep. This indicates a complex interplay between symptoms of ADHD and comorbidity, in that each ADHD symptom domain exert an influence on both stress and sleep through different comorbid symptoms. Specifically, inattention had an effect through internalizing symptoms and hyperactivity/impulsivity through externalizing symptoms. Further, an examination of the paths and the strength of the estimates gives that comorbid symptoms (i.e., emotional symptoms and conduct problems) contribute to elevated perceived stress and sleep problems to a larger degree than core ADHD symptoms do.

In line with some previous findings, perceived stress and poor sleep were related to both emotional and externalizing symptoms [17, 28]. These results are in contrast to studies in younger children, where emotional but not externalizing symptoms increased sleep problems [27, 30]. This again proposes that emotional symptoms and conduct problems affect sleep through different mechanisms. With adolescence, increased independence from parental control may escalate certain types of sleep problems, such as staying up late and not getting enough sleep, in individuals with high levels of conduct problems. Interestingly, peer problems contributed to higher levels of perceived stress but was unrelated to ADHD symptoms. Of note, levels of peer problems were low in both groups and the scale may capture another type of social difficulties than typically noted in individuals with ADHD. For instance, the scale describes being lonely and getting along with adults rather than peers, which is more in line with social difficulties typically described in autism.

### Implications

The results have several implications for clinical work. In parallel to interventions directed at reducing core ADHD symptoms, handle stress, and restore sleep, comorbid symptoms may be acknowledged and targeted as a mean to reduce stress and sleep problems and increase wellbeing. Specifically, as comorbidity may be a major mechanism underpinning stress and sleep problems, mapping and targeting these emotional symptoms and conduct problems as part of standard interventions may increase treatment response. Although not studied systematically, medication was not associated with stress and sleep in the current sample. As such, other types of interventions, such as psychological or pedagogical, focusing on comorbidity and directly on the health-related problems may prove a valuable complement or alternative to medical treatment. Further, pedagogical and psychosocial interventions could be used to reduce school-related stress. Adolescent girls with ADHD seem to at particular risk for elevated perceived stress. In light of the fact that girls’ ADHD is detected later than boys’, our results stress the importance of identifying girls with ADHD at an early age. This would enable early intervention, which possibly could prevent escalation of emotional symptoms and the associated high stress levels.

### Limitations

Although our study has merits, we wish to acknowledge some limitations. All our constructs of interest were measured with ratings. To avoid rater bias, we used parent and self-ratings but no objective measures were available. The use of dual raters may result in lower estimates, but at the same time parents are usually better at rating behavioral symptoms and adolescents at emotional symptoms [42, 43]. Semi-structured interviews to map psychiatric symptoms, sleep diaries, objective measures of sleep pattern, and biomarkers of stress such as cortisol levels could be used as more objective alternatives to ratings. Further, the study relies on cross-sectional data, which rules out any true inferences of causation. That is, although our model, based on theoretical assumptions, propose that comorbid symptoms have a mediating effect on stress and sleep, the reverse could also be true (i.e. that stress and poor sleep exert an effect on emotional symptoms and conduct behavior). Most plausible, there are recursive effects that can only be mapped using a longitudinal design with repeated measures. The ADHD group consisted of individuals who all wished to partake in group interventions (and had no ongoing psychological treatment), which resulted in a larger proportion of females. This female bias in treatment research has previously been reported by Hirvikoski and colleagues [56]. Further, a large proportion of the sample were on stable medication. Regarding diversity, the control sample seem to represent the general ethnical distribution of Swedish adolescents. As for the ADHD sample, we lack data on ethnicity for S1 whereas S2 does not contain individuals with both parents being born outside of Scandinavia. Taken together, the results may not generalize to all individuals with ADHD. Finally, the lack of measures of socio-economic factors and family measures such as conflicts and parental mental health also prevent us from controlling for factors beyond psychiatric symptoms that may have an impact on stress and sleep.

## Summary

Perceived stress and sleep problems should be considered as co-occurring features of ADHD. This is of great concern as chronic stress and sleep problems may have profound effects on virtually all aspects of human functioning. Our findings propose that adolescent girls may be at increased risk for elevated stress, whereas the ADHD group in general had poor sleep. These health-related problems seem to be mediated by emotional symptoms and conduct problems, why interventions that are tailored based on the individual pattern of comorbidity and associated stress and sleep issues may be of great importance. In conclusion, the interplay between ADHD symptoms, comorbid symptoms, and health-related issues such as perceived stress and poor sleep is intricate. Taking these associations into account when developing and providing treatment interventions may increase treatment response. Future studies should take advantage of a longitudial design with repeated measures to examine reciprocal effects over time.
